# Magnesium inference screw supports early graft incorporation with inhibition of graft degradation in anterior cruciate ligament reconstruction

**DOI:** 10.1038/srep26434

**Published:** 2016-05-23

**Authors:** Pengfei Cheng, Pei Han, Changli Zhao, Shaoxiang Zhang, Xiaonong Zhang, Yimin Chai

**Affiliations:** 1 Department of Orthopaedic, Shanghai Jiao Tong University Affiliated Sixth People’s Hospital, Shanghai 200233, China; 2 State Key Laboratory of Metal Matrix Composites, School of Materials Science and Engineering, Shanghai Jiao Tong University, Shanghai 200240, China; 3 Suzhou Origin Medical Technology Co. Ltd., Suzhou 215513, China

## Abstract

Patients after anterior cruciate ligament (ACL) reconstruction surgery commonly encounters graft failure in the initial phase of rehabilitation. The inhibition of graft degradation is crucial for the successful reconstruction of the ACL. Here, we used biodegradable high-purity magnesium (HP Mg) screws in the rabbit model of ACL reconstruction with titanium (Ti) screws as a control and analyzed the graft degradation and screw corrosion using direct pull-out tests, microCT scanning, and histological and immunohistochemical staining. The most noteworthy finding was that tendon graft fixed by HP Mg screws exhibited biomechanical properties substantially superior to that by Ti screws and the relative area of collagen fiber at the tendon-bone interface was much larger in the Mg group, when severe graft degradation was identified in the histological analysis at 3 weeks. Semi-quantitative immunohistochemical results further elucidated that the MMP-13 expression significantly decreased surrounding HP Mg screws with relatively higher Collagen II expression. And HP Mg screws exhibited uniform corrosion behavior without displacement or loosening in the femoral tunnel. Therefore, our results demonstrated that Mg screw inhibited graft degradation and improved biomechanical properties of tendon graft during the early phase of graft healing and highlighted its potential in ACL reconstruction.

Anterior cruciate ligament (ACL) reconstruction with a tendon graft fixed by interference screws to bone tunnels is the most broadly accepted procedure for young and active patients who experience ACL tears in modern sports^1^,^2^. This reconstruction procedure has the best chance for success because the graft undergoes extensive biological remodeling and incorporation in bone tunnels^3^,^4^. However, 10–25% of patients develop graft failure and become candidates for revision, especially in the initial phase of rehabilitation^5^,^6^. Graft failure in early rehabilitative activities is characterized by increased pain, a loss of motion, increased pathologic anterior laxity and functional instability of the knee joint^7^,^8^. Revision ACL reconstruction is challenging, and satisfactory results are obtained 3–4 times less frequently compared with primary ACL reconstruction^9^. Therefore, special attention must be taken in the primary ACL reconstruction to avoid a secondary revision.

Graft failure in the initial phase of rehabilitation is commonly related to complicated biological and biomechanical changes in the transformation from tendon grafts into a newly functional ACL. The major histological changes are extended necrosis, collagen degradation and an influx of inflammatory cells in the center of the graft^10^,^11^. Several cytokines are released in the degradation process, and a cascade of growth factors is essential to guide the subsequent remodeling procedures, such as revascularization, cell proliferation and differentiation^12^. However, the inflammatory reaction during the early graft degradation may become an unlimited process in some cases, and these changes may lead to an antagonistic environment for ACL reconstruction, which ultimately causes the lack of sufficient graft incorporation during the early graft healing phase^13^. Therefore, methods to inhibit early graft degradation have been the focus of research to promote tendon graft incorporation and avoid graft failure in the early and aggressive rehabilitation of knee joints.

Magnesium (Mg) materials have recently been the focus of biodegradable implants in musculoskeletal tissues^14^. Mg has mechanical properties that provide rigid fixation of tendon graft and sustain a loading environment^15^. The corrosion rate of Mg devices is adjustable to meet the graft healing^16^, and its corrosion products have bioactivities that promote regeneration of both hard and soft musculoskeletal tissues^17^. Furthermore, biodegradable Mg particles^18^ and implants^19^ have the potential to elicit an inflammatory response *in vivo*. Ezechieli *et al*.^20^ implanted Mg alloy (MgYREZr, which has a composition similar to WE43) pins into the intercondylar femoral notch of a rabbit model and there was no significant inflammatory reaction in the synovial membrane or synovial fluid. Thus, it is essential to determine the influence of biodegradable Mg implants on graft degradation as well as its function in graft fixation during early graft healing process after ACL reconstruction.

The purpose of this study was to investigate the application of Mg interference screws in ACL reconstruction. We hypothesized that the novel interference screw could inhibit graft degradation, promote tendon-bone healing and provide rigid fixation of the tendon graft in the early phase after ACL reconstruction. In the present study, high-purity magnesium (HP Mg) screws were used to fix semitendinosus autografts in a rabbit model of ACL reconstruction with pure titanium (Ti) screws as the control. The influence of Mg screws on graft degradation and the related mechanism was elucidated as well as the corrosion behaviors of Mg screws in the early phase of tendon-graft healing.

## Results

All rabbits returned to daily activity following ACL reconstruction surgery. The surgical intervention and implanted devices were well tolerated with no sepsis, emphysema or wound dehiscence. Moreover, there was no obvious articular degeneration or synovitis surrounding the bone tunnel (Supplementary Fig. 1).

### HP Mg screws promoted biomechanical properties of the reconstructed ACL

**Figure 1:**
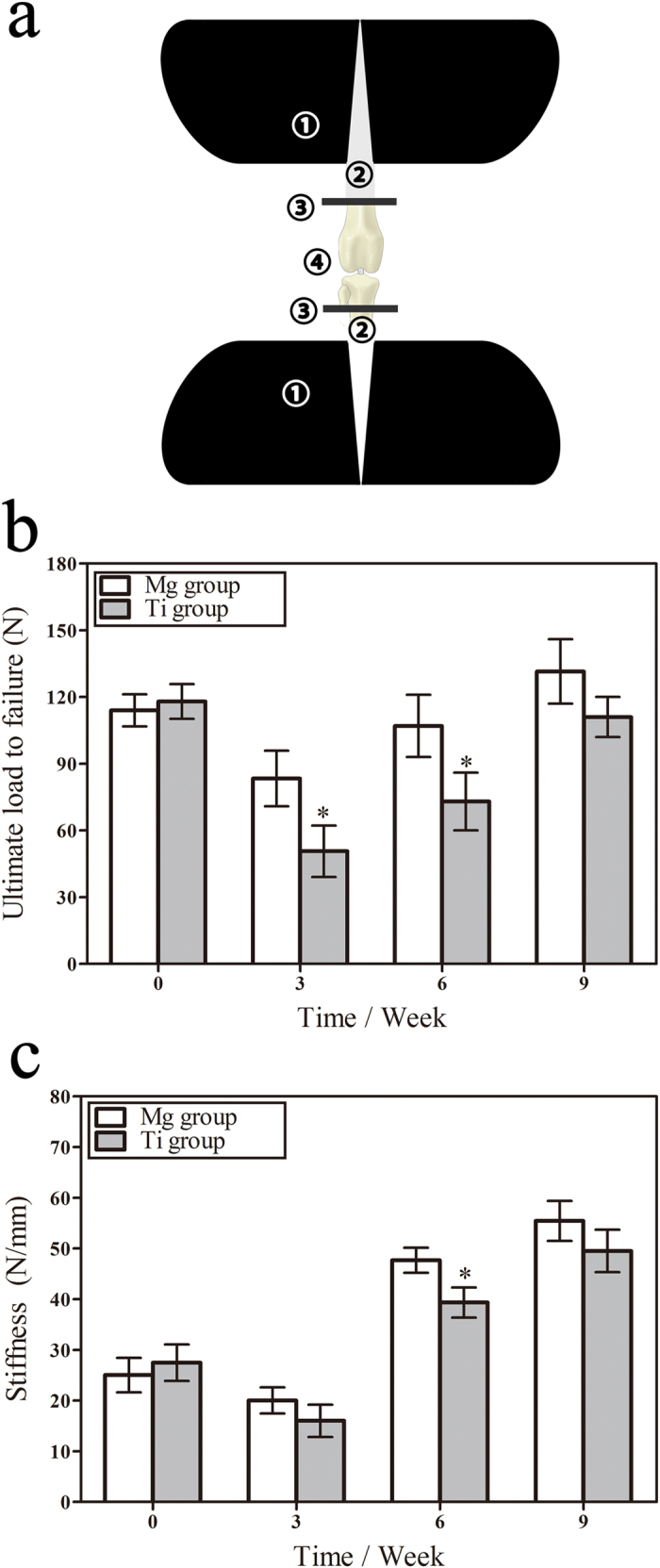
The devices for pull-out tests and the analysis of biomechanical properties of the reconstructed ACL. (**a**) Schematic drawing of pull-out tests (1) device base to reduce motion in measurement; (2) special fixture parallel to the axial direction of the femur-tendon graft-tibia complex; (3) inserted pin for fixation of (4) femur-tendon graft-tibia complex. (**b**) Ultimate load to failure of reconstructed ACL at zero, three, six and nine weeks after surgery. (**c**) Stiffness of reconstructed ACL at failure at zero, three, six and nine weeks after surgery. *denotes p < 0.05.

The biomechanical properties of the reconstructed ACL were reflected in the pull-out tests as in Fig. 1. The failure mode of tendon graft in the pull-out tests showed the weak link of reconstructed ACL during graft healing stages. The ultimate load to failure of tendon graft was determined by both the fixation devices and its biological attachment. It reflected whether the reconstructed ACL could match the requirement in daily activities and a progressive rehabilitation program. The stiffness (N/mm) was calculated from the slope of the linear region of the load deformation curve. When a graft and its fixation device were loaded with a tensile force, displacement in the fixation device and tendon graft was equal to the amount described by its stiffness.

All tendon graft failed by direct pull-out from the femoral tunnel at 0 week. And the HP Mg screw and the Ti screw kept in original position during the pulling-out tests. At this time point, the average ultimate load to failure and stiffness of the Mg group was similar to the Ti group, which was 116 ± 7.2 N and 26.2 ± 3.5 N/mm, respectively. It indicated that initial fixation strength provided by the HP Mg screw was comparable to the Ti screw.

The insertion site of tendon graft appeared to be the weak link of the reconstruction at three weeks. Tendon graft failed by interligamentous ruptures at the portion close to the screw cap, which might be caused by cumulative tissue damage at the screw edge under cyclical loading of daily activities. The ultimate load to failure and the related stiffness of both groups significantly decreased, which elucidated the high morbidity of graft failure during early rehabilitation. At this time point, the ultimate load to failure and stiffness of the Mg group (83.3 ± 12.5 N and 20.3 ± 2.6 N/mm, respectively) were significantly higher than that of the Ti group (50.7 ± 11.5 N and 16.0 ± 3.2 N/mm, respectively).

Direct interference screw fixation of tendon graft with HP Mg screws additionally alters the mechanical properties during its early remodeling, as both the Mg group and the Ti group exhibited an increase in the ultimate load to failure and related stiffness at six weeks. And the midsubstance portion of the graft close to the screw cap represents the weak link. Furthremore, the Mg group remained superior to the Ti group (p < 0.05). The increase in the biomechanical properties of the tendon graft continued at nine weeks, when there was no significant difference detected between two groups. So we concluded that the initial mehcanical strength provided by the HP Mg screw was comparable to the Ti screw, furthermore, the biomechanical properties of tendon graft fixed by the HP Mg screw was superior to that by the Ti screw during the early phase of graft healing. From the biomechanical tests, we found that direct fixation with HP Mg interference screw could withstand unrestricted motion and full loads in the rabbit model, and reconstructed ACLs performed good biomechanical properties.

### Degradation behavior and quantitative analysis of HP Mg screw *in vivo*

**Figure 2:**
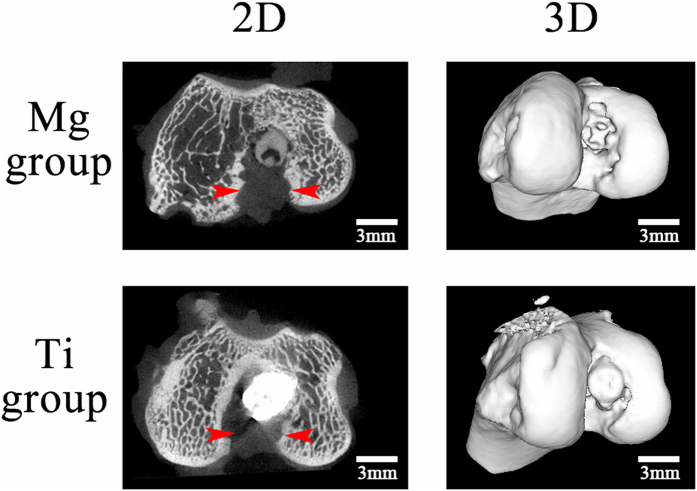
MicroCT view of the interference screws in fixation of tendon graft in femoral intracondyle. MicroCT 2D and 3D views indicated that the HP Mg and Ti screws were deployed in the original position at nine weeks post-operation, and the semitendinosus tendon grafts (red arrowheads) were rigidly fixed in the femoral tunnel.

The *in vivo* behavior of HP Mg screws was assessed via high resolution microCT. Nine weeks after surgery, both the HP Mg screw and Ti screw were deployed at the original position without displacement or loosening (Fig. 2). The tendon graft was rigidly fixed in the femoral tunnels. Furthermore, no obvious gas accumulation was identified in the bone tissue that surrounded the interference screw or underneath the soft tissue.

**Figure 3:**
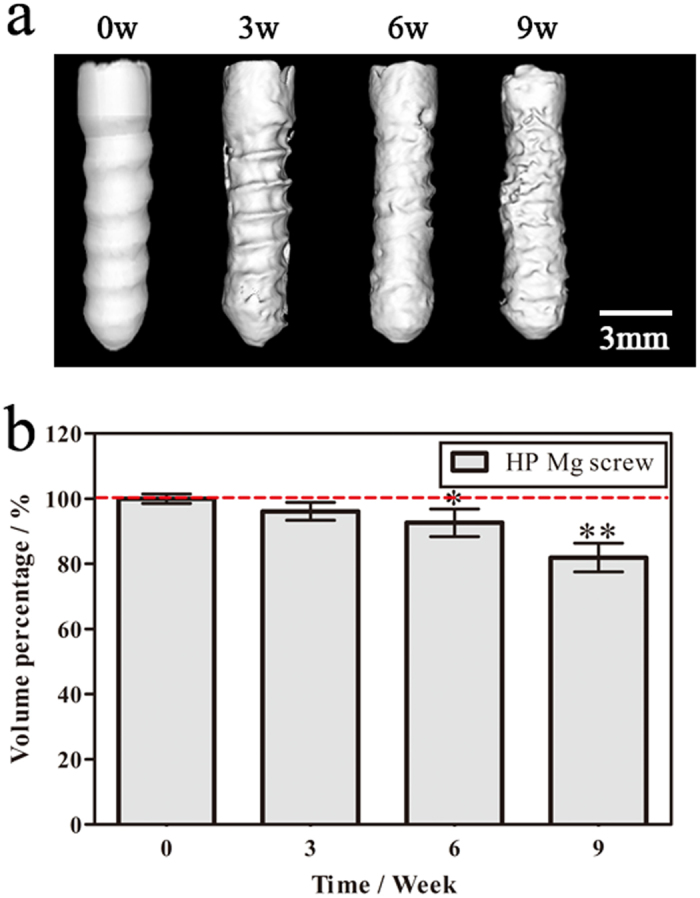
Morphology and volume loss of the Mg screw *in vivo*. MicroCT 3D morphology (**a**) and volume (**b**) of the HP Mg screw *in vivo* prior to surgery and three, six and nine weeks after surgery.

As biodegradable implants, the HP Mg screws exhibited uniform corrosion behavior *in vivo*, and the morphological changes in different regions of HP Mg screws were uniform (Fig. 3). The screw thread became flattened, and the thread depth decreased with time. Moreover, mineral deposition on the screw surface was visible at nine weeks. In the calculation of the screw volume, the HP Mg screw remained 81.9 ± 4.4% of the initial volume after nine weeks. The whole volume of the HP Mg screw decreased by 10.4 ± 2.7 mm^3^, and the corrosion rate was 0.165 mm^3^/day.

### Graft degradation and remodeling in the early phase of graft healing

**Figure 4:**
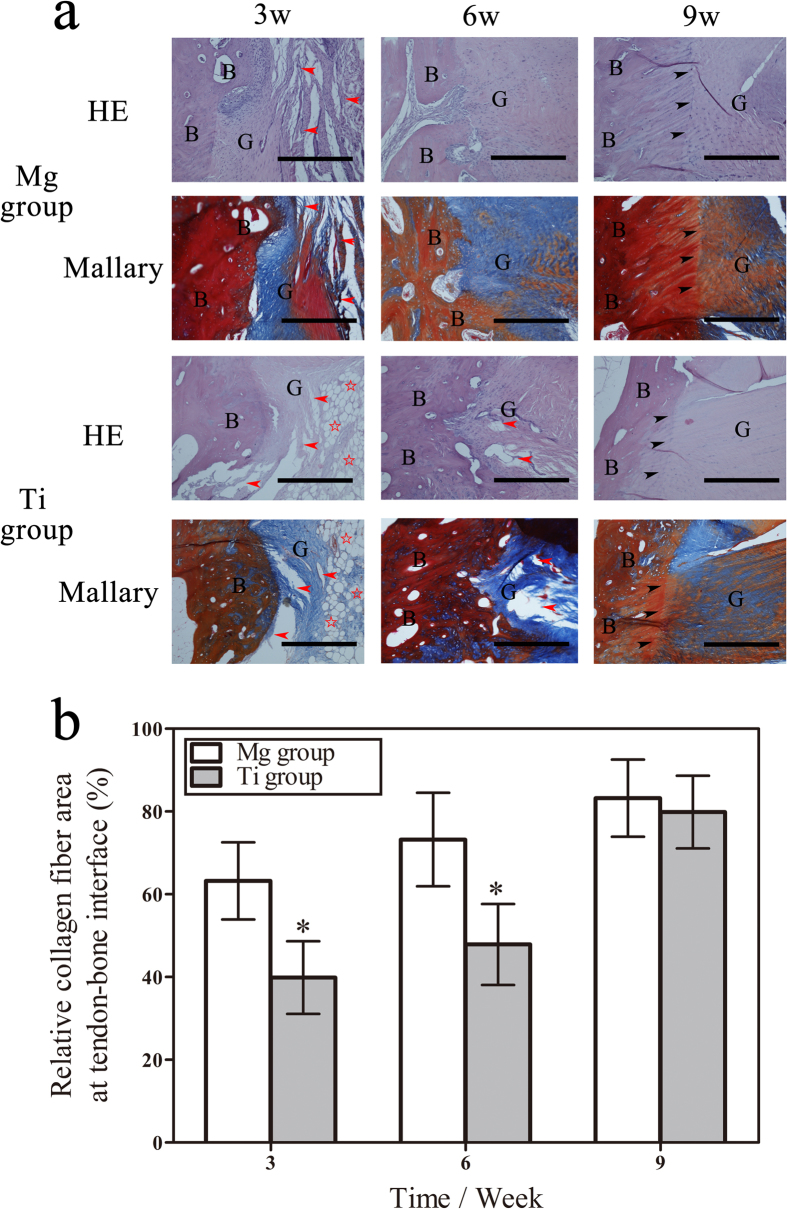
Degradation and remodeling of tendon graft fixed by the HP Mg screw and Ti screw. (**a**) Representative sections of the tendon-bone interface at three, six and nine weeks after surgery. HE and Mallary stained. G represents the tendon graft, B represents bone, red arrowheads represent defragmented collagen fibers, red pentagram represents the necrosis of collagen fibers, and black arrowheads represent Sharpey-like fibers. Bar = 400 μm. (**b**) Calculated relative area of collagen fiber at the tendon-bone interface. *denotes p < 0.05.

The histological images at the tendon-bone interface reflected graft degradation in the initial phase of graft healing (Fig. 4). At three weeks, the histological changes were characterized by collagen degradation. The collagen bundles of the Mg group were sparsely distributed with a cleavage of collagen molecules. In comparison, the graft at the tendon-bone interface of the Ti group exhibited more collagen defragment, splitting or even necrosis. Furthermore, the relative area of the collagen fiber at the tendon-bone interface was significantly increased in the Mg group compared with the Ti group (P = 0.0075). These findings indicated that the graft degradation of the Mg group was relatively inhibited, and the Ti group was more severe than the Mg group in the graft degradation during early graft healing.

At six weeks, collagen tissues proliferated and were aligned to the bone. The regenerated collagen fibers of the Mg group refilled the interface and were densely arranged. In contrast, the collagen tissues of the Ti group were sparsely distributed and irregularly adhered to the interface, and several cavities were identified in the collagen fibers. In the quantitative assessment, the Mg group maintained its advantage in the relative area of collagen fiber at the tendon-bone interface compared with the Ti group.

The collagen fibers at the tendon-bone interface remodeled at nine weeks. In the ligamentization process, newly formed collagen fibrils dominated the tendon graft, and these Sharpey-like fibers were in direct apposition with the bone. The occurrence of highly differentiated Sharpey-like fibers in the Mg group indicated maturation of graft healing, while the differentiation of collagen fibers in the Ti group was relatively low. Furthermore, the relative area of collagen fiber at the tendon-bone interface substantially increased at nine weeks, and there was no significant difference between the Mg group and the Ti group in the relative collagen fiber area at the tendon-bone interface.

### Expression of MMP-13 and Collagen II at the tendon-bone interface

**Figure 5:**
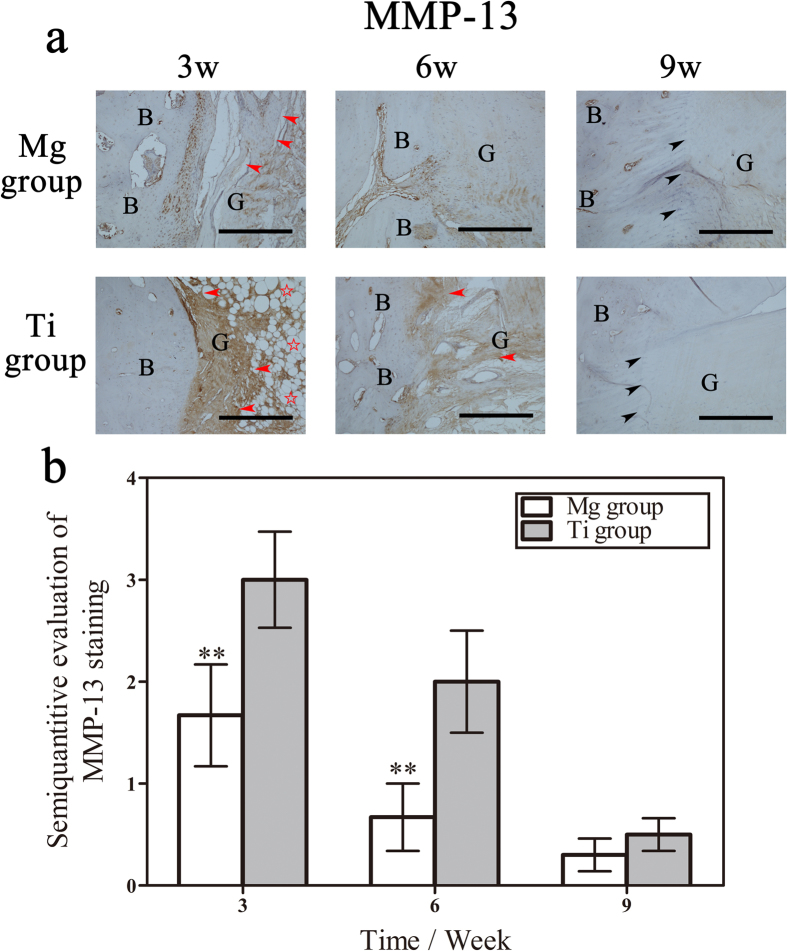
Distribution and quantitative analysis of MMP-13 at the tendon-bone interface. (**a**) Representative photomicrographs showing the immunohistochemical staining of MMP-13 at the tendon-bone interface three, six and nine weeks after surgery. G represents the tendon graft, B represents bone, red arrowheads represent defragmented collagen fibers, the red pentagram represents the necrosis of collagen fibers, and black arrowheads represent Sharpey-like fibers. Bar = 400 μm. (**b**) Semi-quantitative evaluation of MMP-13 immunohistochemical staining. **denotes p < 0.01.

MMP-13 comprised the collagen degradation related proteinase. The positive staining of MMP-13 at the tendon-bone interface reached a peak at three weeks and subsequently subsided (Fig. 5). And the MMP-13 expression at nine weeks was hardly detected. These findings indicated that MMP-13 was active in the initial phase of graft healing. Furthermore, the expression of MMP-13 in the Ti group was increased compared with the Mg group at both three and six weeks, which was consistent with the relatively severe collagen degradation surrounding the Ti screw compared with that surrounding the HP Mg screw.

**Figure 6:**
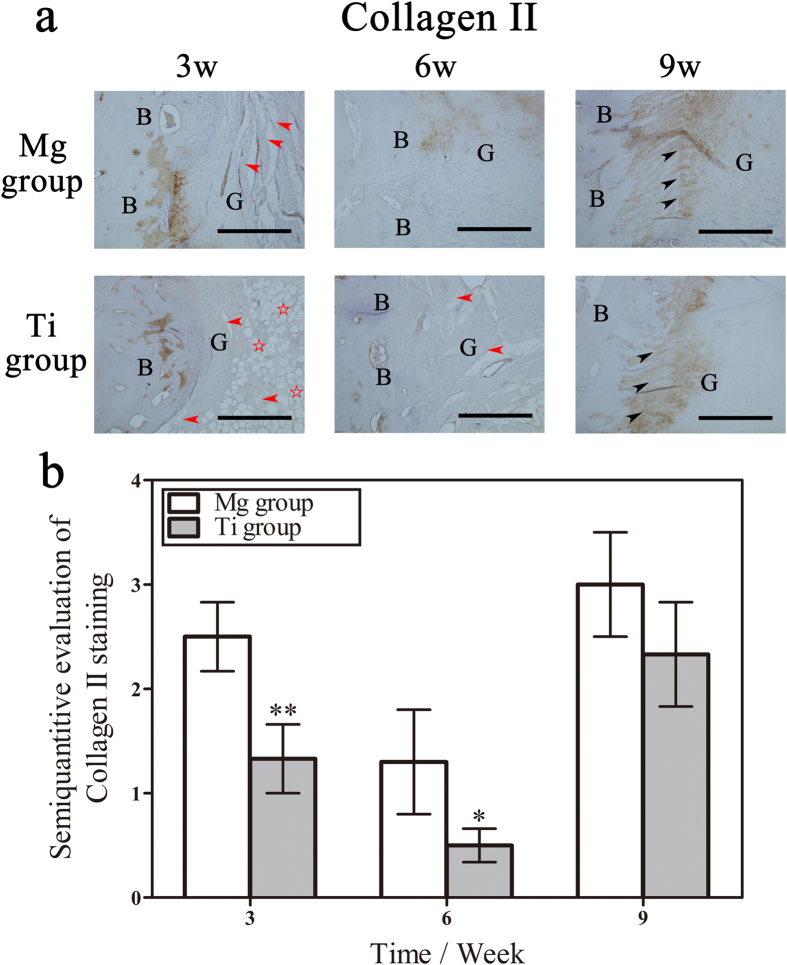
Distribution and quantitative analysis of Collagen II at the tendon-bone interface. (**a**) Representative photomicrographs showing the immunohistochemical staining of MMP-13 at the tendon-bone interface three, six, nine and twelve weeks after surgery. G represents the tendon graft, B represents bone, red arrowheads represent defragmented collagen fibers, the red pentagram represents the necrosis of collagen fibers, and black arrowheads represent Sharpey-like fibers. Bar = 400 μm. (**b**) Semi-quantitative evaluation of MMP-13 immunohistochemical staining. **denotes p < 0.01.

Collagen II was specifically expressed at the tendon-bone interface of matured native ACLs and was the specific substrate to MMP-13 proteinase. Highly positive staining of Collagen II was identified at three weeks postoperatively and decreased at six weeks (Fig. 6). These findings indicated the remnant collagen of the native ACLs was in degradation during the early phase of graft healing. In this process, the positive staining of Collagen II was relatively stronger in the Mg group compared with the Ti group. The positive staining of Collagen II subsequently increased at nine weeks, which indicated the regeneration of matured ACL collagen fibers at the tendon-bone interface.

## Discussion

The present study identified the potential of Mg interference screws in ACL reconstruction, especially during the initial phase of graft healing. HP Mg screws provided rigid mechanical strength comparable to traditional Ti screws in the fixation of the tendon graft; thus, the femur-tendon graft-tibia complexes of both groups failed with equal valence at 0 week. As biodegradable materials, the mechanical retention of Mg interference screws was directly related to the corrosion rate^21^, and HP Mg screws exhibited stable corrosion *in vivo* at approximately 0.165 mm^3^/day. Furthermore, during the early graft healing period, the corrosion products of HP Mg screws promoted the biological fixation of the tendon graft. At three and six weeks post-surgery, the HP Mg screws supported a relative larger area of the collagen fiber at the tendon-bone interface compared with the Ti screws. The Mg group also exhibited higher ultimate load to failure and related stiffness of the tendon graft during this period. In contrast, the tendon graft that surrounded the Ti screws exhibited significantly lower biomechanical properties and relative area of the collagen fiber at the interface. The improvement in the biomechanical properties of reconstructed ACL by HP Mg screw was critical, as it could prevent graft failure during early rehibilitation activities. Current rehabilitation stresses immediate full range of motion and early weight bearing within six weeks after ACL reconstruction^22^, but the weak link of graft fixation hindered the early and aggressive rehabilitation^23^,^24^. HP Mg screws supported rigid and stiff biological fixation, especially at 3 and 6 weeks, and therefore allowed current rehabilitation principles of early and aggressive rehabilitation, which will greatly benefits the early return of knee joint function.

HP Mg screws supported rigid biological fixation of tendon graft likely due to its influence in graft degradation. It was widely accepted, that the time period of early graft healing was marked by graft degradation and an ensuing inflammatory response. According to previous report^25^,^26^, no graft revascularization had been established shortly after surgery, thus increasing collagen disintegration and hypocellularity was identified at the tendon bone interface. Shortly afterwards, neutrophils and recruited macrophages were triggered by the graft necrosis, and these cells produced cytokines, including MMP-13 and transforming growth factor-β (TGF-β), which were essential for the reconstruction of tendon grafts following surgery^27^. The inflammatory phase was critical for the following remodeling of the tendon graft. Extensive studies have reported that the severe inflammatory reaction could lead to the decrease in the biomechanical properties of the tendon graft or even graft failure in the phase of graft healing^3^,^4^,^28^. Current data in present study also elucidated the weak attachment of tendon grafts during the early postoperative period, which exhibited collagen degradation, defragment and necrosis of the tendon graft at the interface. The weak attachment of tendon grafts during early graft healing was reflected in the biomechanical parameters, as the ultimate load to failure and related stiffness simultaneously decreased with structural changes at 3 and 6 weeks. Within these observation time point, the Mg group performed advantages in biomechanical properties of reconstructed ACL. Furthermore, the Mg group also showed rare collagen necrosis cavities or defragmentation at the tendon-bone interface. These histological findings elucidated how the HP Mg screws inhibited graft degradation and improved the biomechanical properties of reconstructed ACLs. Although the differences between the Mg group and the Ti group in both biomechanical properties and relative area of the collagen fiber became negligible at 9 weeks, the inhibition of graft degradation by the Mg screw during early graft healing led to the regeneration of highly differentiated Sharpey-like fibers in direct apposition with the bone. Sharpey-like fibers was the characteristics in collagen remodelling stage, and it was critical for the healing graft toward the morphology and mechanical strength of the intact ACL, which enhanced graft stability and long-term clinical outcome^26^.

**Figure 7:**
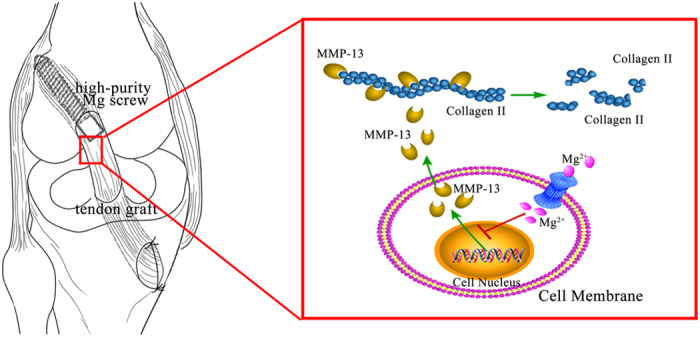
Schematic diagram delineating the blockage of MMP-13 and Collagen degradation by Mg ion at the tendon-bone interface. HP Mg screw fixed the tendon graft to the femoral tunnel in ACL reconstruction. Mg ion was released during the screw corrosion, went through the ion channel and became absorbed by the native tissues at the tendon-bone interface. The intra-cellular Mg ion blocked the MMP-13 expression, inhibited the degradation of collagen II and reserved collagen fibers at the tendon-bone interface during graft degradation. Part of this figure refers to the figures in our previous work (ref. 48) and the permission to resuse the figures has been got.

In this study, we confirmed that the corrosion products of HP Mg screws modulated the expression of MMP-13 and played critical roles in graft degradation. MMPs comprise a family of zinc-dependent proteases, which are active in degrading extracellular matrix proteins, i.e. collagen and elastin, and maintain the dynamic homeostasis of extracellular microenvironments^29^,^30^,^31^. Of the MMPs, MMP-13 is the unique interstitial collagenase in connective and cartilaginous tissues, which is frequently monitored in a number of pathological conditions, such as ligament tears and tendon injury^32^,^33^. Several reports have confirmed the inhibition of graft degradation and enhancement of ACL grafts incorporation via MMP-13 blockage. Demirag and colleagues decreased the MMP-13 activity in a rabbit ACL reconstruction model with recombinant-alpha-2-macroglobulin (A2 M) protein and identified a significantly higher load to failure and a greater number of Sharpey-like fibers at the tendon-bone interface^34^. Antimicrobial agents (doxycycline^35^ and fluoroquinolones^36^) and proinflammatory cytokines (TNF-α^37^ and IL-1β^38^) with inhibitive effects on MMP-13 have also been reported to promote tendon-bone healing in research. Our present study confirmed that MMP-13 was active at the tendon-bone interface during the early graft healing and participated in the collagen degradation of the tendon graft. Mg interference screws inhibited the expression and wide distribution of MMP-13 at the tendon-bone interface based on immunohistochemical analysis. Furthermore, Collagen II, the specific substrate to MMP-13, was identified in relatively higher expression at the interface that surrounded HP Mg screws three and six weeks after surgery, indicating the native collagen tissues at the interface preserved in graft degradation process under the influence of Mg. Therefore, We hypothesized that the inhibitory effects of HP Mg screws on graft degradation might occur in this way, that the Mg ion released from corroded HP Mg screws went through the ion channel, blocked the MMP-13 expression, inhibited the degradation of collagen II and preserved collagen fibers at the tendon-bone interface (Fig. 7).

The signaling pathways activated by Mg ions and led to MMP-13 blockage were highlighted in many researches. According to previous research, the inhibition of MMPs by Mg products likely occurred via tyrosine kinase inhibitors. Ueda *et al*. and Toyoda *et al*. demonstrated that extracellular Mg dose-dependently reduced MMP production in cultured rat vascular smooth muscle cells as well as rat cardiac fibroblasts potentially via two tyrosine kinase inhibitors, genistein and herbimycin A^39^,^40^,^41^. In clinical trials, intravenous administration of Mg ion in patients with acute myocardial infarction and reperfusion reduced the production of MMP-1 and protected myocardial cells from reperfusion injuries^42^,^43^. However, the exact mechanism involved in MMP-13 inhibition by Mg in ACL reconstruction was still unclear.

In conclusion, Mg screws exhibited substantial potential in the tendon graft fixation of ACL reconstruction. First, tendon grafts fixed by HP Mg screws exhibited a relatively higher biomechanical strength compared with traditional Ti screws. Second, HP Mg screws exhibited a larger area of collagen fibers at the tendon-bone interface early after ACL reconstruction, which was critical for the biological fixation of the tendon graft. Third, HP Mg screws reduced the MMP13 expression at the tendon-bone interface during the early phase of graft healing and maintained more Collagen II in the collagen degradation, which elucidated the mechanism involved in the inhibition of collagen degradation and graft failure by Mg screws.

## Methods

### Material preparation

**Figure 8:**
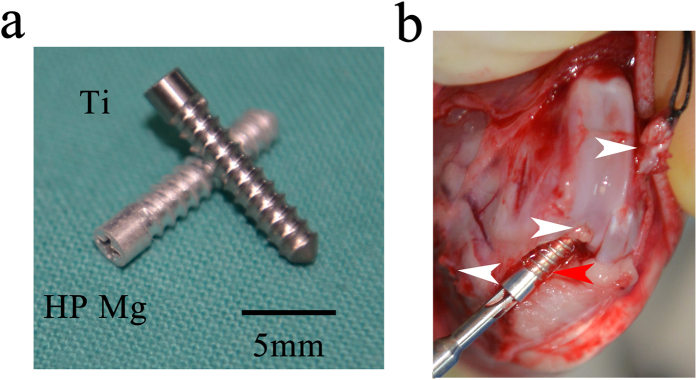
Microscopic view of the HP Mg and Ti screws and their application in the reconstruction of ACL. (**a**) HP Mg and Ti screws exhibited the same design. The screws were 12 mm in length, 2.1 mm in core diameter and 2.7 mm in major diameter. (**b**) Macroscopic view of ACL reconstruction procedure. Semitendinosus (white arrowhead) was fixed to the femoral tunnel by HP Mg screws (white arrowhead).

HP Mg screws (99.98 wt.%) and Ti screws (TA1ELI, 99.8 wt.%) were supplied by Suzhou Origin Medical Technology Co. Ltd., China. As shown in Fig. 8a, both the HP Mg and Ti screws had the same design, and the parameters (length 12 mm, core diameter 2.1 mm, major diameter 2.7 mm) were set according to rabbit knee joint geometry. Prior to implantation, all screws were cleaned with acetone and demineralized water to remove residual debris; the screws were subsequently sterilized with 29 kGy of ^60^Co radiation.

### Surgery procedure

The animal experiments were approved by the Animal Care and Experiment Committee of Shanghai Jiao Tong University Affiliated Sixth People’s Hospital. All experimental protocols were conformed to the Guide for the Care and Use of Laboratory Animals (NIH Publication Eighth Edition, updated 2011). The ACL reconstruction surgery was performed on the right leg of 60 skeletally matured, male, New Zealand white rabbits. In brief, following exposure of the knee joint space through medial parapatella arthrotomy, the original ACL was completely transected. The semitendinosus was subsequently freed from the proximal end. With the rabbit knee in 45° flexion, a bone tunnel with a diameter of 2.1 mm was drilled through the tibia to the femoral condyle as shown in Fig. 8b. The semitendinosus was pulled through both the femoral and tibial tunnels and subsequently fixed to the femoral tunnel with an interference screw. The distal end of the semitendinosus was sutured on the periosteum at the exit of the tibial tunnel. Afterwards, the wound was irrigated and closed in layers.

Following recovery from anesthesia, the rabbits were maintained in individual cages and allowed to eat and drink ad libitum. Antibiotic (Amoxicillin 150 mg/10 kg weight) was subcutaneously administered for three days. The wound healing process and daily activities after surgery were recorded during the experimental period.

### Retrieval of the femur-tendon graft-tibia complex

Three, six and nine weeks after surgery, the rabbits were killed. Femur-tendon graft-tibia complexes were harvested with 60 mm of femur and 60 mm of tibia. Soft tissues adjacent to the tendon graft were clearly removed. Gross observation was performed regarding articular degeneration and tendon graft incorporation on the articular surface.

### Biomechanical tests

For the biomechanical properties of the femur-tendon graft-tibia complexes, pull-out tests were conducted at zero, three, six and nine weeks following surgery. The left leg of the rabbit model was performed ACL reconstruction surgery immediately following euthanasia and was used as the 0 week group in the biomechanical tests. Two bone ends of the femur-tendon graft-tibia complex were fixed in a Zwick materials testing machine (Z020, Zwick/Roell, Germany) using inserted pins (Fig. 2). The axial side of the complex was parallel to the direction of the applied loads with a preloading of 1 N. The load displacement rate was set at 0.5 mm/min. The failure modes of the tendon graft, the ultimate load to failure (N) and the related stiffness (N/mm) at each time point were recorded according to reference^44^.

### Micro-computed tomography (microCT) evaluation

MicroCT scanning of the femur-tendon graft-tibia complexes was conducted at three, six and nine weeks post-surgery. The MicroCT device (Laboratory Micro-CT Scanner eXplore RS 80, GE Healthcare, Little Chalfont, UK) was set as follows: 80 kV and 450 μA in the X-ray tube, a scan resolution of 45 μm and an exposure time of 400 ms. The volume of the P Mg screw was analyzed using Micro View 2.2 Advanced Bone Analysis Application software (GE Health Systems, Waukesha, WI, USA). The percentage volume change (Δv) was calculated as the difference between the initial volume (v0) and the final volume (v1):


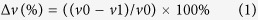


Three samples were scanned for each type of interference screw, with an average of three times per sample.

### Histological analysis

Following fixation in 4% neutral buffered formalin for 48 hours, the femur-tendon graft-tibia complex fixed by an HP Mg screw and Ti screw was decalcified in 9% formic acid for nine weeks at room temperatures^45^. The specimen was washed in phosphate-buffered saline solution, dehydrated and subsequently embedded in paraffin. Sections 4-μm thick, which included the tendon graft and femoral bone tunnel, were cut in the coronal plane. Consecutive sections were stained with hematoxylin and eosin (HE) and Mallory tri-chrome. The tendon graft that surrounded the tendon-bone interface was observed via optical microscopy (Leica DM2500, Leica, Germany) at ×40.

The graft degradation was determined by the relative collagen fiber area at the tendon-bone interface. The range of interest (ROI) was localized at the entrance of the femoral tunnel, where the tendon graft was fixed by the screw. The calculation were performed using Image-Pro Plus 6.0 (Media Cybernetics, Rockville, Marland, USA) according to a previous study^46^. In brief, the area of the tendon-bone interface was histomorphometrically quantified by drawing two lines along the most superficial boundaries of the collagen fibers and the longitudinal extension lines of the top newly formed bone. The collagen fiber area was divided by the deepest and the most superficial boundaries along the chondrocytes next to the distal newly formed bone and the proximal tendon. The relative collagen fiber area at the tendon-bone interface was measured by dividing the collagen fiber area with the area of the tendon-bone interface area. Three trained observers analyzed the relative area of the collagen fibers, and three sections were examined per specimen.

### Immunohistochemical analysis

The immunohistochemical staining of matrix metalloproteinase-13 (MMP-13) and Collagen II was performed as subsequently described. In brief, consecutive sections were dewaxed in xylene and then rehydrated through a series of graded alcohols. The endogenous peroxidase activity was subsequently quenched with 0.3% hydrogen peroxide for 10 minutes. After blocking with 1% goat serum (Sigma) (1:100 dilution), the sections were incubated with primary antibodies against MMP-13 (1:200; Abcam, Cambridge, UK) and Collagen II (1:100; Novus Biologicals, Littleton, CO, USA) overnight at 4 °C. Following washing in PBS, the sections were incubated in secondary antibody for 1 h at 37 °C. The staining was developed in 3,3′-diaminobenzidine (DAB) solution (Dako, Hamburg, Germany) with hematoxylin counterstaining. Immunohistochemical analysis of the stained sections was subsequently performed under optical microscopy at ×40. The semi-quantitative scoring of the immunohistochemical sections was also measured using an image analyzer (Image-Pro Plus, Media Cybernetics, USA) and a five-grade scoring system (Supplementary Table 1) adopted from Lovric *et al*.^47^.

### Statistical analysis

All data are presented as the means ± standard deviations. Statistical analysis was performed using the SPSS 17.0 software package (SPSS Inc., Chicago, USA). T-tests determined the level of significance in the differences, and p values less than 0.05 and 0.01 were considered significant and highly significant, respectively.

## Additional Information

**How to cite this article**: Cheng, P. *et al*. Magnesium inference screw supports early graft incorporation with inhibition of graft degradation in anterior cruciate ligament reconstruction. *Sci. Rep.* **6**, 26434; doi: 10.1038/srep26434 (2016).

## Supplementary Material

Supplementary Information
