# Supplementary material for: Magnesium inference screw supports early graft incorporation with inhibition of graft degradation in anterior cruciate ligament reconstruction
Source: Sci Rep. 2016 May 23;6:26434. doi: 10.1038/srep26434 (PMC4876376; doi:10.1038/srep26434)
Supplement: Supplementary Information [file srep26434-s1.pdf]

SUPPLEMENTARY INFORMATION for

Magnesium inference screw supports early graft incorporation with inhibition of graft degradation in anterior cruciate ligament reconstruction

Pengfei Cheng[1], Pei Han[1], Changli Zhao[2,*], Shaoxiang Zhang[2,3], Xiaonong Zhang[2,3,*], and Yimin Chai[1,*]

1 Orthopaedic Department, Shanghai Jiao Tong University Affiliated Sixth People's Hospital, Shanghai 200233, China. 2 State Key Laboratory of Metal Matrix Composites, School of Materials Science and Engineering, Shanghai Jiao Tong University, Shanghai 200240, China. 3 Suzhou Origin Medical Technology Co. Ltd., Suzhou 215513, China

* Corresponding authors.
Yimin Chai, M.D., Ph.D.,
Department of Orthopaedic, Shanghai Jiao Tong University Affiliated Sixth People's Hospital, Shanghai Jiao Tong University, Shanghai 200233, China.
Phone/Fax: 86-21-6436-9181; E-mail: chaiyimin@vip.163.com

Changli Zhao, Ph.D.,
State Key Laboratory of Metal Matrix Composites, School of Materials Science and Engineering, Shanghai Jiao Tong University, Shanghai 200240, China.
Phone/Fax: 86-21-3420-2759; E-mail: zcl@sjtu.edu.cn

Xiaonong Zhang, Ph.D.,
State Key Laboratory of Metal Matrix Composites, School of Materials Science and Engineering, Shanghai Jiao Tong University, Shanghai 200240, China.
Phone/Fax: 86-512-5229-6383; E-mail: xnzhang@originmedtech.com

SUPPLEMENTARY FIGURES

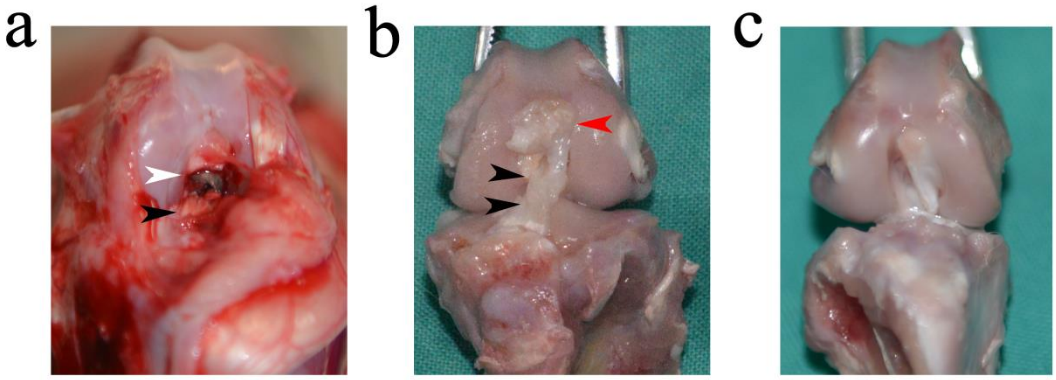

Supplementary Figure S1. **Morphology changes of reconstructed ACL in rabbit femoral intracondylae.** (a) Macroscopic view of reconstructed ACL during surgery. Semitendinosus (black arrowhead) was fixed to femoral tunnel by HP Mg screws (white arrowhead). (b) Macroscopic view of femur-tendon graft-tibia complex at nine weeks after surgery (Red arrowhead marks synovial sheath). (c) Macroscopic view of femur- native ACL -tibia complex.

SUPPLEMENTARY TABLES

Supplementary Table S1. **Five-grade scoring system for immunohistochemical evaluation.**

Items		Score
Percentage of positively stained cells versus whole cell population	Staining intensity	
0%	No	0
＜10%	Weak	1
＜25%	Strong	2
＜50%	Moderate	2
＜50%	Strong	3
＜80%	Moderate	3
＞80%	Strong	4
